# Establishment and Validation of an Individualized Cell Cycle Process-Related Gene Signature to Predict Cancer-Specific Survival in Patients with Bladder Cancer

**DOI:** 10.3390/cancers12051146

**Published:** 2020-05-02

**Authors:** Run Shi, Xuanwen Bao, Paul Rogowski, Christian Schäfer, Nina-Sophie Schmidt-Hegemann, Kristian Unger, Shun Lu, Jing Sun, Alexander Buchner, Christian Stief, Claus Belka, Minglun Li

**Affiliations:** 1 Department of Radiation Oncology, University Hospital, LMU Munich, D-81377 Munich, Germany; Run.Shi@med.uni-muenchen.de (R.S.); paul.Rogowski@med.uni-muenchen.de (P.R.); Christian.Schaefer@med.uni-muenchen.de (C.S.); Nina-Sophie.Hegemann@med.uni-muenchen.de (N.-S.S.-H.); unger@helmholtz-muenchen.de (K.U.); Jing.Sun@med.uni-muenchen.de (J.S.); Claus.Belka@med.uni-muenchen.de (C.B.); 2 Technical University of Munich, D-80333 Munich, Germany; xuanwen.bao@tum.de; 3 Research Unit Radiation Cytogenetics, Helmholtz Center Munich, German Research Center for Environmental Health GmbH, D-85764 Neuherberg, Germany; 4 Department of Radiotherapy, Sichuan Cancer Hospital & Institute, Sichuan Cancer Center, School of Medicine, University of Electronic Science and Technology of China, Chengdu 610041, China; lushun1982@live.cn; 5 Department of Urology, University Hospital, LMU Munich, D-81377 Munich, Germany; Alexander.Buchner@med.uni-muenchen.de (A.B.); Christian.Stief@med.uni-muenchen.de (C.S.)

**Keywords:** bladder cancer, cell cycle process, gene signature, cancer-specific survival, therapeutic resistance

## Abstract

More accurate models are essential to identify high-risk bladder cancer (BCa) patients who will benefit from adjuvant therapies and thus helpful to facilitate personalized management of BCa. Among various cancer-related hallmarks and pathways, cell cycle process (CCP) was identified as a dominant risk factor for cancer-specific survival (CSS) in BCa. Using a series of bioinformatic and statistical approaches, a CCP-related gene signature was established, and the prognostic value was validated in other independent BCa cohorts. In addition, the risk score derived from the gene signature serves as a promising marker for therapeutic resistance. In combination with clinicopathological features, a nomogram was constructed to provide more accurate prediction for CSS, and a decision tree was built to identify high-risk subgroup of muscle invasive BCa patients. Overall, the gene signature could be a useful tool to predict CSS and help to identify high-risk subgroup of BCa patients, which may benefit from intensified adjuvant therapy.

## 1. Introduction

Bladder cancer (BCa) is a common malignancy in the urological system worldwide, with an estimated 430,000 newly diagnosed cases per year. Among these cases, about two-thirds are non-muscle-invasive bladder cancer (NMIBC), while the rest are classified as muscle-invasive bladder cancer (MIBC) [1]. Despite improved understanding of BCa biology and advances in treatments, outcomes of BCa patients remain suboptimal. For example, the standard treatment for localized MIBC is radical cystectomy with bilateral pelvic lymph node dissection, which provides a 5-year overall survival rate less than 50% [2]. Although Tumor-Node-Metastasis (TNM) staging and pathological grading systems are widely used for cancer management and survival prediction, clinical outcomes remain variable in BCa patients, even with similar characteristics [3]. Hence, establishment of a more precise model is essential for individual patients to identify high-risk subgroup who may benefit from systemic adjuvant therapies.

In recent years, advancements in high-throughput techniques such as microarray and RNA-sequencing (RNA-seq) have provided new insight into transcriptome profiling, highlighting the role of molecule markers in cancer diagnosis and prognosis [4,5]. Several groups have developed gene signatures to predict progression or survival in BCa patients [6,7,8]. However, the exact biological function of each gene in such a gene signature was often not clear, nor the interaction of them. Till now, the clinical utility of these signatures remains limited, and few of them were applied to clinical practice.

In this study, we mined public databases and developed a cell cycle process-related risk score (CCPRS) to predict cancer-specific survival (CSS) in BCa patients, which was further validated in other independent cohorts from Gene Expression Omnibus (GEO) and The Cancer Genome Atlas (TCGA). In combination with clinicopathological features, we aimed at constructing an integrated model to improve predictive power and risk stratification for CSS of individual BCa patients.

## 2. Materials and Methods

### 2.1. Dataset Preparation and Data Processing

Four microarray datasets including 587 BCa patients with clinical annotations and CSS information were downloaded from Gene Expression Omnibus (GEO). GSE13507 was produced by Illumina human-6 v2.0 expression beadchip and was used as the training set in our study. GSE31684 (Affymetrix Human Genome U133 Plus 2.0 Array) and GSE32894 and GSE32548 (Illumina HumanHT-12 V3.0 expression beadchip) were used as independent validation cohorts. Probe IDs were mapped to gene symbols according to the corresponding annotation file, and expression measurements of all probes linking to a same gene were averaged to obtain a single value. RSEM-normalized RNA-seq data, copy number data, and clinical phenotypes of MIBC samples were obtained from The Cancer Genome Atlas (TCGA). Copy number data and TPM data of 22 bladder cell lines were obtained from Cancer Cell Line Encyclopedia (CCLE) [9]. RMA normalized microarray expression data and IC50 values of different drugs for 1018 cell lines were accessed from Genomics of Drug Sensitivity in Cancer (GDSC) [10]. All microarrays and RNA-seq data included in this study were normalized and log2 transformed.

### 2.2. Candidate Selection and Signature Establishment

In brief, the levels of cancer-related hallmarks and pathways in each sample from the training set were quantified by a single-sample gene set enrichment analysis (ssGSEA) [11] algorithm based on the transcriptome profiling data and corresponding gene sets retrieved from Molecular Signatures Database (MSigDB) [12]. Cox proportional-hazards regression model was used to evaluate the risk of each hallmark and pathway for CSS in BCa patients using R package ‘survival’. Weighted correlation network analysis (WGCNA) was used to construct a scale-free co-expression network using R package ‘wgcna’ [13] and identify the module which is mostly correlated with CCP based on transcriptome profiling data and CCP ssGSEA score. Gene significance (GS) represents the association of individual genes with CCP ssGSEA score, and module membership (MM) depicts the correlation between module eigengenes and gene expression profiles. With a threshold of *p* value of GS < 0.0001 and *p* value of univariate Cox regression < 0.0001, 64 candidates from CCP module remained. Subsequently, a least absolute shrinkage and selection operator (LASSO) Cox regression model was used to further screen out the most robust prognostic genes [14]. A detailed screening diagram is shown in Figure S1. A cell cycle process-related risk score (CCPRS) was calculated by including normalized gene expression value weighed by LASSO Cox coefficient as follows:

$$CCPRS=\underset{i}{\sum }Coefficient\left(mRNA_{i}\right)\times Expression\left(mRNA_{i}\right)$$

### 2.3. Additional Bioinformatic and Statistical Analyses

IBM SPSS Statistics 20 (IBM Corp., Armonk, NY, USA), GraphPad Prism 8.0 (GraphPad Software Inc, San Diego, CA, USA), Stata 12 ((StataCorp LLC, College Station, TX, USA), and R software (version 3.5.2, http://www.r-project.org) were used to analyze data and plot graphs. Z-score method was used to normalize ssGSEA scores and CCPRS when necessary. GSEA [15] was performed to confirm the positive regulation role in CCP. Unsupervised hierarchical clustering was performed to show distance between hallmarks and pathways, and a network depicting their relationships and connectivity was generated by the Cytoscape software [16]. Principal coordinates analysis (PCoA) was used to visualize dissimilarity of two groups based on Bray–Curtis distance matrix. Circos plot was used to visualize enrichment results of Gene Ontology analysis and overlapping genes involved in different biological processes. The webtool cBioPortal for Cancer Genomics [17] was used to visualize the genomic alterations of the established gene signature in BCa samples from TCGA. The Kaplan–Meier method was used to draw survival curves, and the log-rank test was performed to evaluate survival difference. Cox proportional-hazards regression model was used to evaluate the significance of each parameter for the risk of CSS. Meta-analysis was performed to evaluate the prognostic value of CCPRS in the pooled cohort. Pearson correlation coefficient is used to evaluate the relationship between two continuous variables with a normal distribution. K-means-based consensus clustering using R package ‘ConsensusClusterPlus’ [18] or non-negative matrix factorization (NMF) consensus clustering [19] using R package ‘nmf’ was performed to obtain subgroups based on a gene expression matrix. Nomogram and calibration analysis were generated using ‘rms’ package. Time-dependent receiver operating characteristic (tROC) analysis was performed to measure the predictive power of the nomogram using ‘survivalROC’ package, and a calibration curve was plotted to visualize the predictive accuracy of the nomogram. Recursive partitioning analysis was performed to construct a decision tree of risk stratification for CSS with R package ‘rpart’ [20]. Student’s *t*-test or one-way analysis of variance (ANOVA) was used to analyze differences between groups in variables with a normal distribution. A *p* value less than 0.05 was considered statistically significant.

## 3. Results

### 3.1. Schematic Diagram of the Study Design

**Figure 1:**
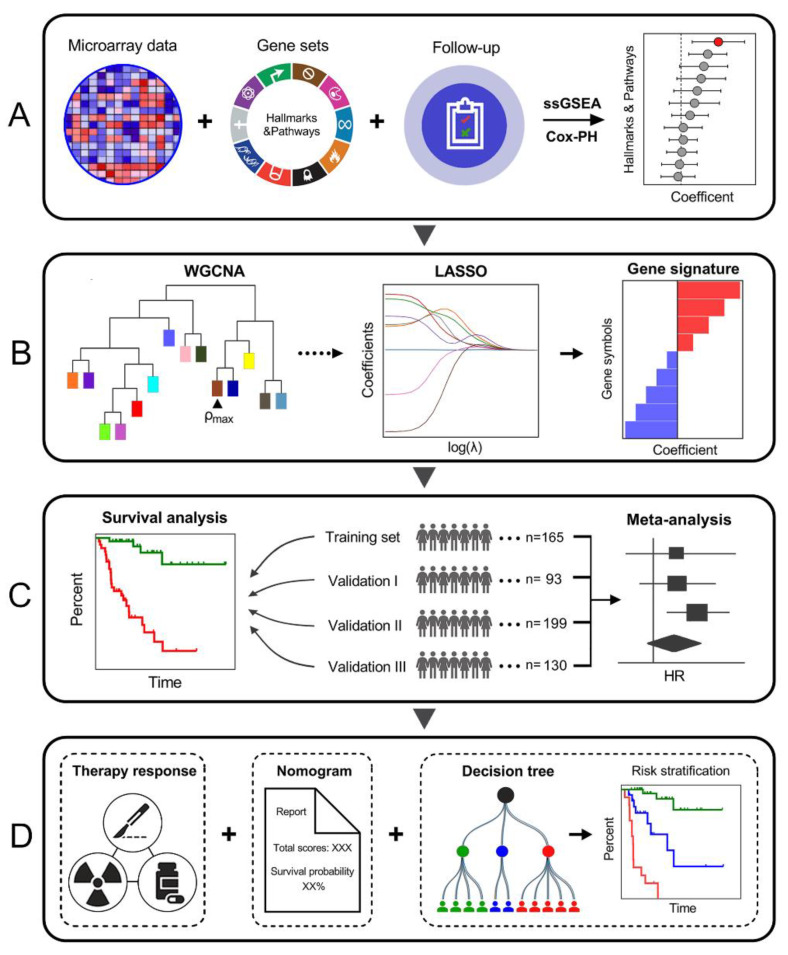
Schematic diagram of the study design. (**A**) Among various cancer-related hallmarks and pathways, cell cycle process (CCP) was identified as the dominant risk factor for cancer-specific survival (CSS) in bladder cancer (BCa) patients. (**B**) Combined methods were used to establish a robust CCP-related gene signature for CSS. (**C**) The prognostic value of the gene signature was further validated in different cohorts. (**D**) Clinical application.

First, cell cycle process (CCP) was identified as the dominant risk factor for CSS in BCa patients among various cancer-related hallmarks and pathways (Figure 1A). Then, WGCNA was performed to identify a CCP module. A series of screening methods including LASSO algorithm were used to screen out most promising candidates and to develop a robust CCP-related gene signature for CSS prediction (Figure 1B). Subsequently, a CCPRS formula was established to quantify risk assessment for BCa patients, and the prognostic value was evaluated in the training and independent validation cohorts. Meta-analysis was performed to evaluate CCPRS in the pooled cohort (Figure 1C). In addition, response to anti-cancer therapies was evaluated to investigate whether the gene signature is a valuable marker for therapeutic resistance. Regarding clinical application, based on the combination of CCPRS and traditional prognostic variables, a nomogram was generated to quantify the risk assessment for individuals and a decision tree was constructed to improve risk stratification for CSS in BCa patients (Figure 1D).

### 3.2. Cell Cycle Process was Identified as the Primary Risk Factor for CSS

**Figure 2:**
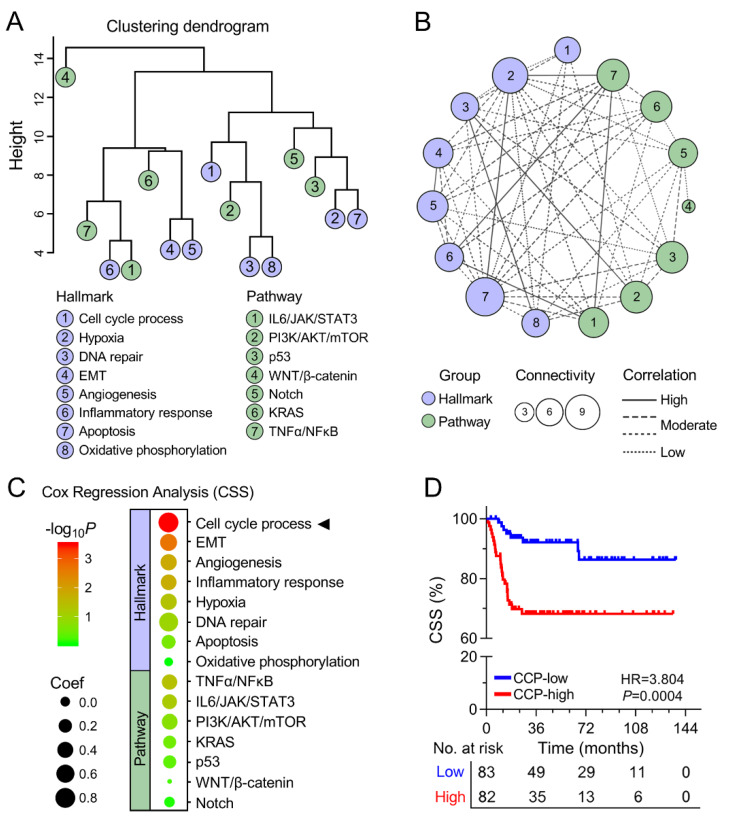
CCP was identified as the primary risk factor for CSS. (**A**) Hierarchical clustering dendrogram was generated to show the distance between different hallmarks and pathways. (**B**) A network was constructed to depict relationships between different hallmarks and pathways. **(C**) Bubble heatmap of Cox results indicated that CCP serves as the most powerful risk factor for CSS. (**D**) Kaplan–Meier curve demonstrated that patients with higher CCP single-sample gene set enrichment analysis (ssGSEA) scores exhibited worse CSS compared to those with lower scores.

The levels of each cancer-related hallmark and pathway were quantified by ssGSEA. A hierarchical clustering dendrogram was generated to show the distance between different hallmarks and pathways, and WNT/β-catenin signaling appeared to be the most distinct (Figure 2A). Moreover, a network depicting their relationships is shown in Figure 2B. The network was constructed based on the soft threshold of connectivity derived from Pearson correlation between any two nodes (coefficient < 0.5 was ignored). In the network, bigger size represented a more significant role, and solid lines represented higher correlation. Subsequently, Cox proportional-hazards regression analysis was performed based on ssGSEA scores of cancer-related hallmarks and pathways and CSS information in the training set, and bubble heatmap indicated that CCP serves as the most powerful risk factor for CSS (Figure 2C). Kaplan–Meier curve demonstrated that patients with higher CCP ssGSEA scores exhibited worse CSS compared to those with lower scores when the median value was the cut-off value (HR = 3.804, 95% CI = 1.893–7.643, *p* = 0.0004; Figure 2D).

### 3.3. Identification of a Cell Cycle Process-Related Gene Module

**Figure 3:**
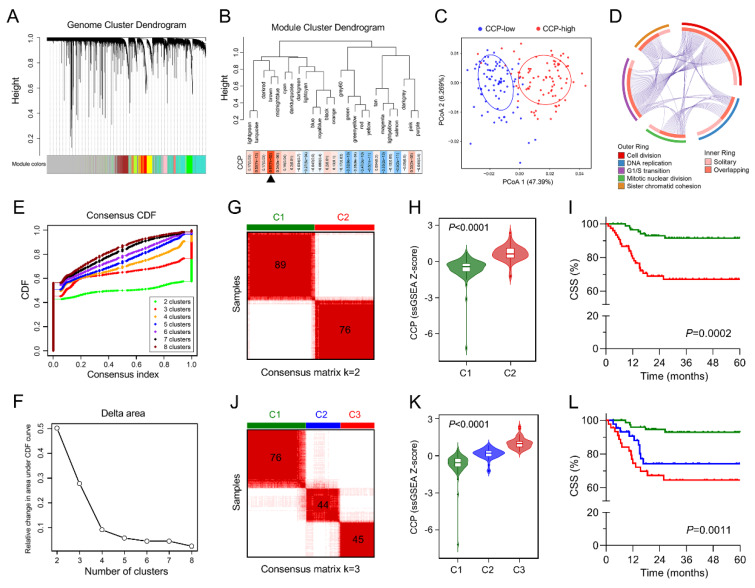
Identification of a CCP-related gene module. (**A**) Weighted correlation network analysis (WGCNA) was performed with transcriptome profiling data and CCP ssGSEA Z-scores in the training set. (**B**) The brown module depicting the highest correlation (r = 0.87, *p* = 7e–52) with CCP ssGSEA scores was considered as “CCP module”. (**C**) Principal coordinates analysis (PCoA) was performed to visualize the dissimilarity of CCP-low and CCP-high samples based on the Bray–Curtis distance matrix. (**D**) Circos plot demonstrated that all of five most significant processes were labelled with cell cycle-related features. (**E**) Based on expression pattern of genes involved in the brown module, K-means-based consensus clustering was performed to classify the training group into different subgroups. Cumulative distribution function (CDF) plot showed the cumulative distribution functions of the consensus matrix for each k (from 2 to 8, indicated by colors). (**F**) The difference between two CDF curves (k and k-1) is illustrated by measuring the area under the CDF curves from k = 2 to 8 in the delta area plot. (**G**,**J**) k = 2 and 3 were chosen as optimal parameters to divide the training cohort into different subgroups. (**H**,**K**) Violin plots showed that CCP ssGSEA scores were differentially distributed in identified subgroups. **(I**,**L**) Patients exhibited different CSS in identified subgroups.

Firstly, WGCNA was performed with transcriptome profiling data and CCP ssGSEA Z-scores in the training set (Figure 3A). Sample clustering showed that one outlier was detected and excluded (Figure S2). A total of 25 non-grey modules were generated with a power of β = 9 as the optimal soft threshold to ensure a scale-free co-expression network (Figure S3). Among these modules, the brown module depicting the highest correlation with CCP ssGSEA scores was considered as “CCP module” (r = 0.87, *p* = 7 × 10^−52^; Figure 3B). The scatter diagram showed a highly positive correlation between GS and MM in the brown module (r = 0.87, *p* < 1 × 10^−200^; Figure S4), indicating this module is highly correlated with CCP. Based on the Bray–Curtis distance matrix derived from the expression pattern of the brown module genes of BCa samples, PCoA was performed to visualize the dissimilarity. We observed that samples of low CCP and high CCP were clearly separated into two discrete groups (Figure 3C). Then, all the 1126 genes involved in the brown module were submitted to Gene Ontology for enrichment analysis. Circos plot demonstrated that five most significant processes were labelled with cell cycle-related features such as cell division, DNA replication, G1/S transition, mitotic nuclear division, and sister chromatid cohesion, with a high proportion of overlapping genes involved (Figure 3D). To further confirm whether the brown module could represent CCP, a K-means-based consensus clustering was performed to classify the training group into different subgroups according to the expression patterns of the 1126 genes involved in the brown module. Cumulative distribution function (CDF) plot showed the cumulative distribution functions of the consensus matrix for each k (from 2 to 8, indicated by colors), which is a quantification of how entries of the consensus matrix are distributed within the range from 0 to 1 (Figure 3E). The difference between two CDF curves (k and k − 1) is illustrated by measuring the area under the CDF curves from k = 2 to 8 in the delta area plot (Figure 3F). Then, we chose k = 2 and 3 as optimal parameters to divide the training cohort into different subgroups (Figure 3G,J). The violin plot showed that CCP ssGSEA scores were significantly elevated in cluster 2 (C2) compared to cluster 1 (C1) (*p* < 0.0001; Figure 3H), and patients in C2 exhibited worse CSS (*p* = 0.0002; Figure 3I). Similar CCP ssGSEA distribution (*p* < 0.0001; Figure 3K) and CSS difference (*p* = 0.0011; Figure 3L) were observed in three different subgroups derived from consensus clustering when k = 3. These results demonstrated that genes involved in the brown module could represent the feature of CCP.

### 3.4. Establishment of a CCP-Related Gene Signature for CSS

**Figure 4:**
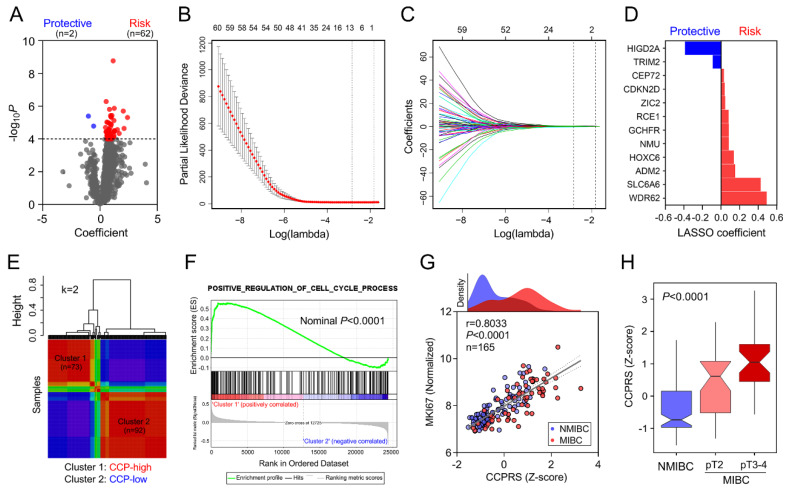
Establishment of a CCP-related gene signature for CSS. (**A**) Volcano plot showed that 64 promising candidates were filtered out. (**B**) Least absolute shrinkage and selection operator (LASSO) algorithm was used to identify the most robust prognostic genes for CSS. Cross-validation was applied to overcome over-fitting effect. (**C**) An optimal λ value of 0.0585 was selected. (**D**) Distribution of LASSO coefficients of the established gene signature. (**E**) Non-negative matrix factorization (NMF) consensus clustering was used to divide the training cohort into two subgroups based on the expression matrix of the established gene signature when k = 2. (**F**) GSEA analysis confirmed the positive regulation role of the gene signature in cell cycle process in BCa samples. (**G**) Cell cycle process-related risk score (CCPRS) was significantly positively correlated with MKI67 in 165 BCa samples (r = 0.8033, *p* < 0.0001). (**H**) CCPRS was significantly elevated in muscle-invasive bladder cancer (MIBC) samples, especially in more advanced stages (*p* < 0.0001).

With a threshold of *p* value for GS less than 0.0001, hub genes from the brown module were submitted to univariate Cox proportional-hazards model. The volcano plot showed that with a threshold of *p* value for Cox regression analysis less than 0.0001, 64 promising candidates were filtered out (Figure 4A). Subsequently, the LASSO algorithm was used to identify the most robust prognostic genes for CSS. Cross-validation was applied to overcome over-fitting effect (Figure 4B), and the optimal λ value of 0.0585 was selected (Figure 4C). An ensemble of 12 genes remained with their individual LASSO coefficients, and the distribution of LASSO coefficients of the gene signature is shown in Figure 4D. NMF consensus clustering was used to divide the training cohort into two subgroups based on the expression matrix of the established gene signature when k = 2 (Figure 4E). According to the expression pattern of the two subgroups, cluster 1 was labeled with CCP-high and cluster 2 as CCP-low. GSEA analysis confirmed the positive regulation role of the gene signature in cell cycle process in BCa samples by comparing cluster 1 and cluster 2 (Nominal *p* < 0.0001; Figure 4F). Considering cell cycle process is tightly correlated with cancer cell proliferation, we investigated the correlation between CCPRS and MKI67 expression. Pearson correlation test indicated that CCPRS was significantly positively correlated with MKI67 in 165 BCa samples (r = 0.8033, *p* < 0.0001; Figure 4G). In addition, CCPRS was significantly elevated in MIBC samples, especially in more advanced stages (*p* < 0.0001; Figure 4H). These results suggested that the gene signature enhanced proliferative ability via positive regulation of CCP in BCa.

### 3.5. Copy Number Alteration is Closely Correlated with Dysregulated Expression of the Gene Signature

**Figure 5:**
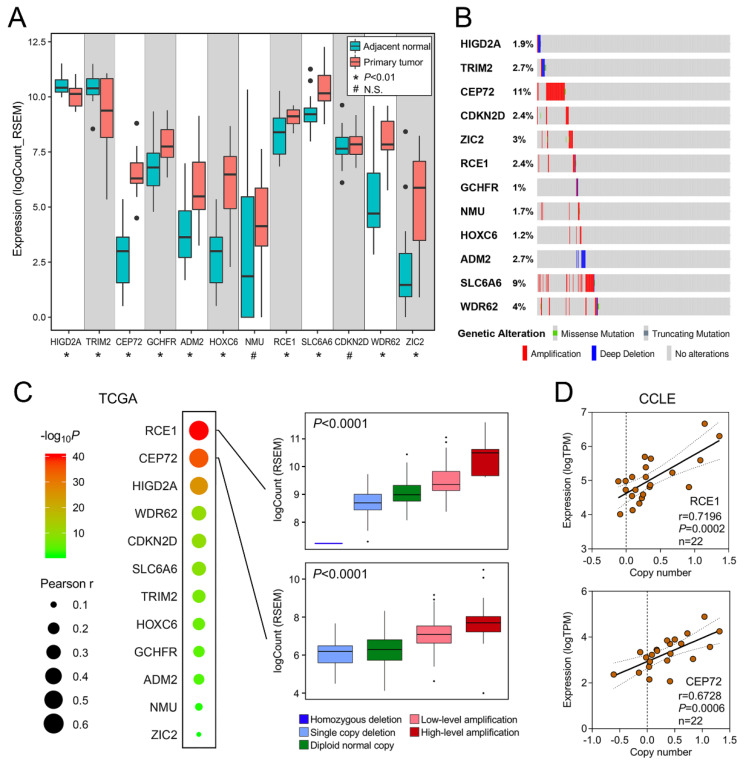
Copy number alteration is closely correlated with dysregulated expression of the gene signature. (**A**) Most genes (10/12) involved in the gene signature were dysregulated in 19 paired BCa and adjacent normal tissues from The Cancer Genome Atlas (TCGA). (**B**) An Oncoprint was plotted to visualize the genomic alterations including mutation and copy number alteration of the gene signature in 413 TCGA BCa samples. (**C**) Overview of relationships between copy number and mRNA expression is plotted. Bubble size represents Pearson correlation coefficient, and color presents the significance. Two representative genes RCE1 and CEP72 with r > 0.5 are shown in detail. (**D**) Copy numbers of RCE1 and CEP72 were frequently amplified in most BCa cell lines, and highly positive correlation between copy number and mRNA expression of RCE1 (r = 0.7196, *p* = 0.0002) and CEP72 (r = 0.6728, *p* = 0.0006) was observed in 22 BCa cell lines. * *p* < 0.01; N.S., no significance.

We investigated the expression profiles of the gene signature in 19 paired BCa and adjacent normal tissues from TCGA. As shown in Figure 5A, paired *t*-test indicated most genes [11,12,13] involved in the gene signature were dysregulated in BCa samples compared to normal. The webtool cBioPortal for Cancer Genomics was used to generate Oncoprint to visualize the genomic alterations including mutation and copy number alteration of the gene signature in 413 TCGA BCa samples. Mutation was rarely detected but copy number alterations of the gene signature frequently occurred in BCa tissues (Figure 5B). We integrated copy number and RNA-seq data from TCGA, and an overview of relationships between copy number and mRNA expression is plotted in Figure 5C. Bubble size represents Pearson correlation coefficient, and color presents the significance. Two representative genes RCE1 and CEP72 with r > 0.5 are shown in detail: mRNA expression values of both genes were progressively and significantly elevated in copy number amplification groups (*p* < 0.0001 for both genes). In addition, copy numbers of RCE1 and CEP72 were frequently amplified in most BCa cell lines, and highly positive correlation between copy number and mRNA expression of RCE1 (r = 0.7196, *p* = 0.0002) and CEP72 (r = 0.6728, *p* = 0.0006) was observed in 22 BCa cell lines (Figure 5D).

### 3.6. Higher CCPRS Predicts Worse CSS in BCa

**Figure 6:**
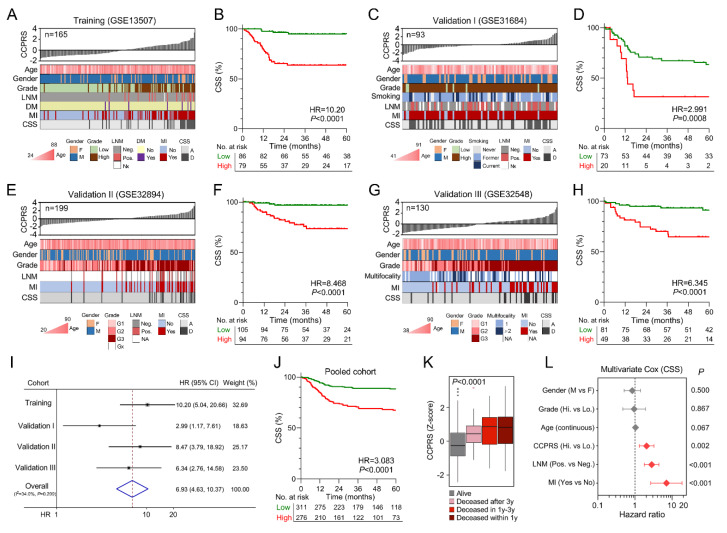
CCPRS correlates with more advanced clinicopathological features and predicts worse CSS. (**A**,**C**,**E**,**G**) Overview of heatmaps depicted associations between CCPRS and different clinicopathological features in each cohort. (**B**,**D**,**F**,**H**) Patients with higher CCPRS exhibited worse CSS in each cohort. (**I**) Meta-analysis on four cohorts included. (**J**) Kaplan–Meier analysis showed that BCa patients with higher CCPRS Z-scores exhibited significantly worse CSS compared to lower scores in the pooled cohort of 587 patients. (**K**) CCPRS Z-scores were significantly elevated in those patients who died during follow-up, with progressively increasing Z-scores as survival time decreased (*p* < 0.0001). (**L**) Multivariate Cox regression analysis on a total of 284 patients with full-scale information including CCPRS, gender, grade, age, lymph node metastasis (LNM) and muscle-invasive (MI) status, and results indicated CCPRS was an independent risk factor for CSS along with MI and LNM.

CCPRS for each sample was calculated and normalized to Z-scores in each cohort. Heatmaps of the associations between CCPRS Z-scores and different clinicopathological features in each cohort were plotted (Figure 6A,C,E,G). In general, an overview of these heatmaps indicated that CCPRS correlates with more advanced clinicopathological features such as muscle-invasive (MI) status and higher grade, while no significant correlation was observed between CCPRS and non-risk factors such as gender. Kaplan–Meier analysis demonstrated that BCa patients with higher CCPRS exhibited worse CSS in each cohort (Training cohort: HR = 10.20, 95% CI = 5.04–20.66, *p* < 0.0001; Validation I: HR = 2.991, 95% CI = 1.175–7.614, *p* = 0.0008; Validation II: HR = 8.468, 95% CI = 3.791–18.92, *p* < 0.0001; Validation III: HR = 6.345, 95% CI = 2.762–14.58, *p* < 0.0001; Figure 6B,D,F,H, respectively). Meta-analysis using fixed-effect model (I^2^ = 34.0%, *p* = 0.209) of the four cohorts showed that higher CCPRS was correlated with a significant worse CSS (pooled HR = 6.93, 95% CI = 4.63–10.37; Figure 6I). Kaplan–Meier analysis also showed that BCa patients with positive CCPRS Z-scores exhibited significant worse CSS compared to negative Z-scores (HR = 3.083, 95% CI = 2.144–4.433, *p* < 0.0001; Figure 6J) in the pooled cohort of 587 patients. In addition, CCPRS Z-scores were significantly elevated in those patients who deceased during follow-up, with progressively increasing Z-scores as survival time decreased (*p* < 0.0001; Figure 6K). Furthermore, as shown in Figure 6L, multivariate Cox regression analysis was performed on a total of 284 patients with full-scale information including gender, grade, age, CCPRS, lymph node metastasis (LNM), and MI status. Results indicated that CCPRS was an independent risk factor for CSS (HR = 2.038, 95% CI = 1.291–3.218, *p* = 0.002) along with MI and LNM.

### 3.7. The Gene Signature Serves as A Promising Marker for Therapeutic Resistance

**Figure 7:**
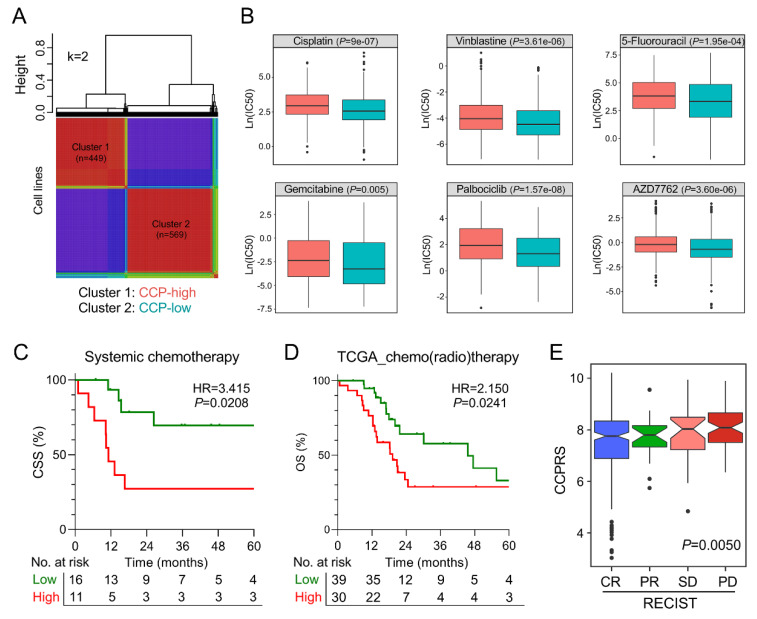
The gene signature serves as a promising marker for therapeutic resistance. (**A**) NMF consensus clustering was performed to divide 1018 cell lines into two clusters. (**B**) IC50 values of different routine chemotherapeutic drugs and cell cycle-targeting drugs were significantly elevated in the NMF-identified CCP-high cluster. (**C**) Among BCa patients who received systemic chemotherapy in the training cohort, those with higher CCPRS exhibited worse CSS. (**D**) Among TCGA MIBC patients who received adjuvant therapies including chemo- or/and radiotherapy, those with higher CCPRS exhibited worse overall survival. (**E**) After initial treatment, CCPRS was progressively and significantly elevated in groups with worse outcomes (*p* = 0.0050).

NMF consensus clustering was performed to divide 1018 cell lines into two clusters based on the gene signature expression matrix when k = 2, and cluster 1 was identified as CCP-high cluster according to the distribution of expression pattern (Figure 7A). We observed that IC50 values of different routine chemotherapeutic drugs (including cisplatin, vinblastine, 5-fluorouracil, and gemcitabine) were significantly elevated in cluster 1 compared to cluster 2. In addition, cell lines in cluster 1 exhibited a significant increased resistance to cell cycle-targeting drugs including palbociclib (CDK4, 6) and AZD7762 (CHEK1, 2) (Figure 7B). Among BCa patients who received systemic chemotherapy in the training cohort, those with higher CCPRS exhibited worse CSS (HR = 3.415, 95% CI = 1.064–10.96, *p* = 0.0208; Figure 7C). Among TCGA MIBC patients who received adjuvant therapies including chemo- or/and radiotherapy, those with higher CCPRS exhibited worse overall survival (HR = 2.150, 95% CI = 1.082–4.270, *p* = 0.0241; Figure 7D). Then we investigated the association between CCPRS and Response Evaluation Criteria in Solid Tumors (RECIST) among TCGA patients. After initial treatment, CCPRS was progressively and significantly elevated in groups with worse outcomes (*p* = 0.0050; CR: complete remission, PR: partial remission, SD: stable disease, PD: progressive disease; Figure 7E).

### 3.8. Combination of CCPRS and Clinical Variables Improves Risk Assessment and Stratification

**Figure 8:**
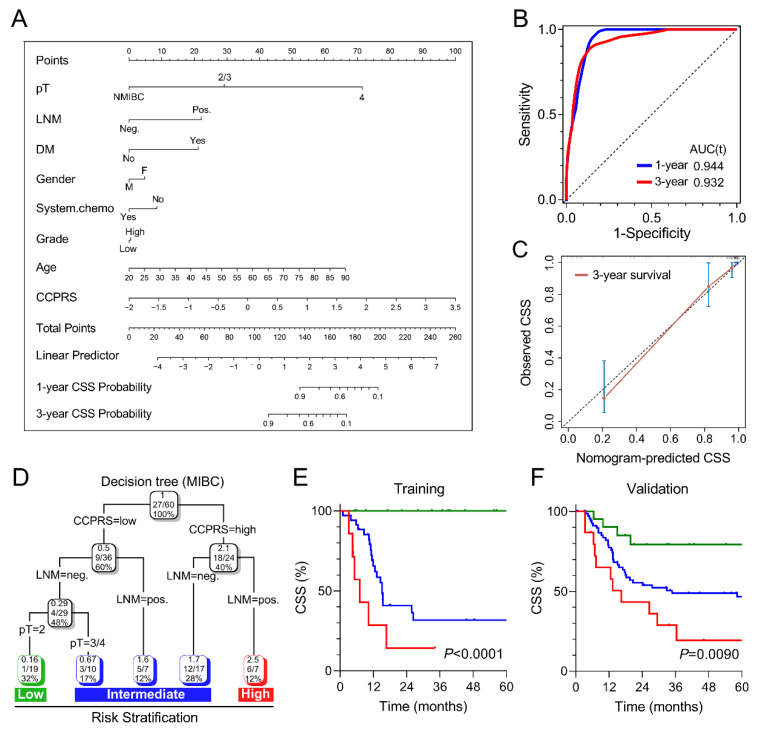
Combination of CCPRS and clinical variables to improve risk assessment and stratification. (**A**) A nomogram was constructed to quantify risk assessment and predict CSS probability for individual patients. (**B**) Time-dependent ROC analysis was performed to evaluate the predictive power of the nomogram for 1-year and 3-year CSS. (**C**) Calibration analysis indicated the nomogram has a high accuracy of CSS prediction. (**D**) An integrated decision tree was generated to optimize the risk stratification for MIBC patients. (**E**) In the training cohort, MIBC patients in the high-risk group exhibited worst CSS compared to other groups (*p* < 0.0001). (**F**) Significant difference of CSS was observed among different risk subgroups in the validation cohort.

To quantify risk assessment and predict CSS probability for individual BCa patients, a nomogram was constructed in combination of CCPRS and clinicopathological features (Figure 8A). The predictive power of nomogram was evaluated using time-dependent ROC analysis, with AUC of 0.944 for 1-year CSS and 0.932 for 3-year CSS, respectively (Figure 8B). In the calibration analysis, the prediction of 3-year CSS closely matched the observed situation, suggesting the nomogram has a high accuracy of CSS prediction (Figure 8C).

Considering MIBC accounts for a considerable part and contributes to the major mortality in BCa, we sought to optimize the risk stratification for MIBC patients by integrating CCPRS with traditional prognostic parameters. Various parameters including age, gender, LNM, grade, pT, and CCPRS were submitted for recursive partitioning analysis. Finally, CCPRS along with LNM and pT remained in the decision tree for CSS, and three different risk subgroups were defined (Figure 8D). Patients in the high-risk group exhibited worst CSS compared to other groups (*p* < 0.0001; Figure 8E). Furthermore, other MIBC patients with full-scale information of CCPRS, LNM, and pT were used to validate the classifying capacity of the decision tree. Significant differences of CSS from GEO (*p* = 0.0090; Figure 8F) and overall survival from TCGA (*p* = 0.0002; Figure S5) were observed among different risk subgroups. These results demonstrated the excellent classifying capacity of the decision tree.

## 4. Discussion

Alteration of cell cycle process (CCP) acts as a critical hallmark of cancer [21,22]. Dysregulation of CCP usually results from a series of changes in the activity of cell cycle regulators, which induces uncontrolled cell division and contributes to cancer development and progression. Abnormalities in expression and amplification of some important CCP-related genes or regulators such as cyclin D1, E1, and E2F1 were frequently observed in various cancer types. Furthermore, it was widely reported that these genomic changes usually promote malignant phenotypes and predict unfavorable outcomes [23,24,25]. In bladder cancer, cell cycle alteration was reported to have prognostic value and some genes involved in CCP have become attractive therapeutic targets [26].

Since Cuzick et al. reported an established cell cycle progression score in prostate cancer in 2011 [27], many studies have followed this approach and validated its prognostic value in different prostate cancer cohorts [28]. In brief, Cruzick’s score is calculated upon the relative expression levels of 31 selected CCP genes normalized to 15 housekeeper genes using quantitative RT-PCR. However, some shortcomings are inevitable in this method. First, these 31 genes, selected from a set of documented CCP genes using Pearson’s correlation coefficient, seem to hardly represent the hallmark of CCP comprehensively. Second, the results of quantitative RT-PCR are quite susceptible to several subjective factors such as different RNA extraction methods, inconsistent human operations, and heterogeneous samples quality in repeated experiments. In this study, ssGSEA algorithm was used to quantify CCP and other cancer-related hallmarks and pathways in BCa samples. ssGSEA calculates separate enrichment scores for each pairing of a sample and gene set, and ssGSEA score represents the degree to which the genes in a particular gene set are coordinately up- or downregulated within a sample. Obviously, this algorithm could overcome shortcomings mentioned above.

Among various cancer-related hallmarks and pathways, CCP was identified as the dominant risk factor for cancer-specific survival (CSS) in BCa. Subsequently, WGCNA was performed to identify CCP-related gene module based on transcriptome profiling data and CCP ssGSEA score. Consensus clustering was used to evaluate whether the identified “CCP module” could represent CCP. Univariate and LASSO Cox regression analyses were successively used to filter out most robust prognostic biomarkers to establish a CCP-related gene signature. The risk score derived from CCP-related gene signature is named as CCP-related risk score (CCPRS) in our study. The prognostic value of CCPRS was evaluated in the training and other independent validation cohorts across different platforms. Moreover, patients with higher CCPRS exhibited worse outcomes in the adjuvant therapy groups, suggesting CCPRS could serve as a useful marker for resistance to anti-cancer treatments. Regarding clinical application, a nomogram was constructed in combination of CCPRS and clinicopathological features to quantify risk assessment and predict CSS probability for individual patients. Considering the significant proportion and high mortality of MIBC in bladder cancer, we combined CCPRS with traditional prognostic parameters to build a decision tree to optimize risk stratification for CSS of MIBC patients.

Most biomarkers involved in the gene signature are dysregulated in BCa compared to normal tissues, and some of them have also been studied in other cancer types. For example, ADM2, one risk biomarker in the gene signature, was reported to predict poor survival in patients with pancreatic adenocarcinoma [29]. Moreover, Wang et al. reported that ADM2 could enlarge the vascular lumen by inducing the quiescent endothelial cell proliferation [30]. Deletion of RCE1 reduced the growth of fibroblasts and skin carcinoma cells [31], and overexpression of RCE1 was reported to correlate with prostate cancer progression and predict poor prognosis [32]. ZIC2, a zinc finger transcription factor, was required for the self-renewal maintenance of liver cancer stem cells, and its depletion reduced sphere formation and xenograft tumor growth in mice [33]. Recessive WDR62 mutations were identified in severe brain malformations [34], and the interaction between WDR62 and mitotic kinase AURKA is essential for drosophila brain growth [35]. What is more, overexpression of WDR62 is associated with poor prognosis in lung adenocarcinoma and gastric cancer [36,37]. In summary, evidence from current literature suggested that some biomarkers involved in the gene signature are closely related to cell proliferation or tumor growth, but the putative role in CCP still needs further investigation.

Some limitations in our study should be acknowledged. First, this is a retrospective study, so the prognostic robustness and clinical usefulness of the gene signature need further validation in prospectively designed clinical trials. Second, further experimental studies are needed to reveal the regulatory role of the gene signature in BCa progression.

## 5. Conclusions

In summary, a novel CCP-related gene signature was established to predict CSS in BCa patients. The prognostic value of CCPRS was further validated in independent cohorts. In combination of the gene signature and clinicopathological features, a nomogram was constructed to quantify risk assessment for individual patients, and a decision tree was built to optimize risk stratification for CSS of MIBC patients. We hope the novel CCP-related gene signature could be a useful tool to select high-risk BCa patients who may benefit from adjuvant therapies and contribute to personalized management of BCa.

## Supplementary Materials

The following are available online at https://www.mdpi.com/2072-6694/12/5/1146/s1, Figure S1. A detailed screening diagram to establish a robust gene signature for CSS; Figure S2. Sample clustering showed one outlier was detected and excluded in WGCNA; Figure S3. A power of β = 9 was chosen as the optimal soft threshold to ensure a scale-free co-expression network; Figure S4. Scatter diagram showed a highly positive correlation between GS and MM in the brown module (r = 0.87, p <1 × 10–200); Figure S5. Significant difference of overall survival in TCGA MIBC patients (*p* = 0.0002) was observed among different risk subgroups defined by the decision tree.

## Abbreviations

BCa	Bladder cancer
CCP	Cell cycle process
CSS	Cancer-specific survival
MI	Muscle invasive
TNM	Tumor-Node-Metastasis
GEO	Gene Expression Omnibus
TCGA	The Cancer Genome Atlas
CCLE	Cancer Cell Line Encyclopedia
GDSC	Genomics of Drug Sensitivity in Cancer
ssGSEA	single-sample gene set enrichment analysis
LASSO	least absolute shrinkage and selection operator
WGCNA	Weighted correlation network analysis
GS	Gene significance
MM	Module membership
CCPRS	Cell cycle process-related risk score
LNM	Lymph node metastasis
RECIST	Response Evaluation Criteria in Solid Tumors
CR	Complete remission
PR	Partial remission
SD	Stable disease
PD	Progressive disease
NMF	Non-negative matrix factorization
tROC	Time-dependent receiver operating characteristic
