# Supplementary material for: Establishment and Validation of an Individualized Cell Cycle Process-Related Gene Signature to Predict Cancer-Specific Survival in Patients with Bladder Cancer
Source: Cancers (Basel). 2020 May 2;12(5):1146. doi: 10.3390/cancers12051146 (PMC7281226; doi:10.3390/cancers12051146)
Supplement: Supplementary file 1 [file cancers-12-01146-s001.pdf]

Supplementary Materials: Establishment and Validation of an Individualized Cell Cycle Process-Related Gene Signature to Predict Cancer-Specific Survival in Patients with Bladder Cancer

Run Shi, Xuanwen Bao, Paul Rogowski, Christian Schäfer, Nina-Sophie Schmidt-Hegemann, Kristian Unger, Shun Lu, Jing Sun, Alexander Buchner, Christian Stief, Claus Belka and Minglun Li

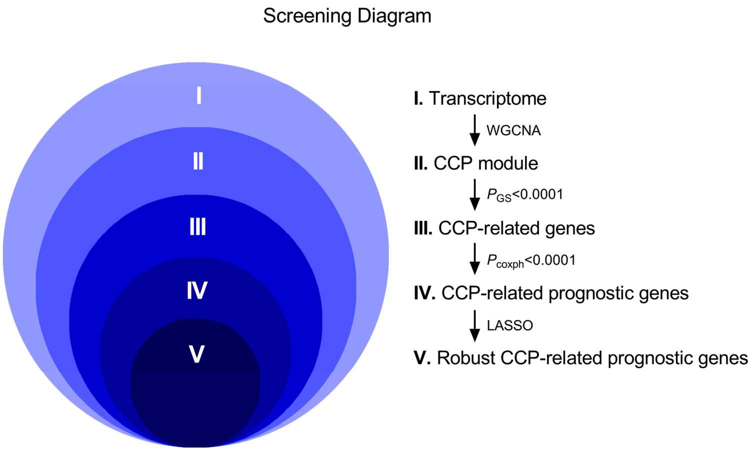

Figure S1. A detailed screening diagram to establish a robust gene signature for CSS.

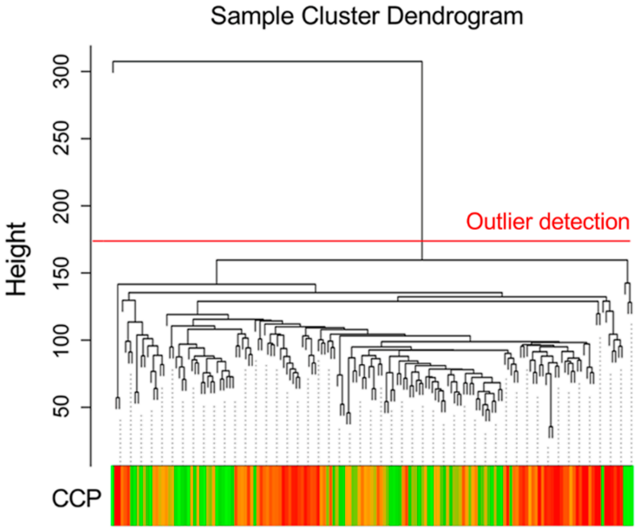

Figure S2. Sample clustering showed one outlier was detected and excluded in WGCNA.

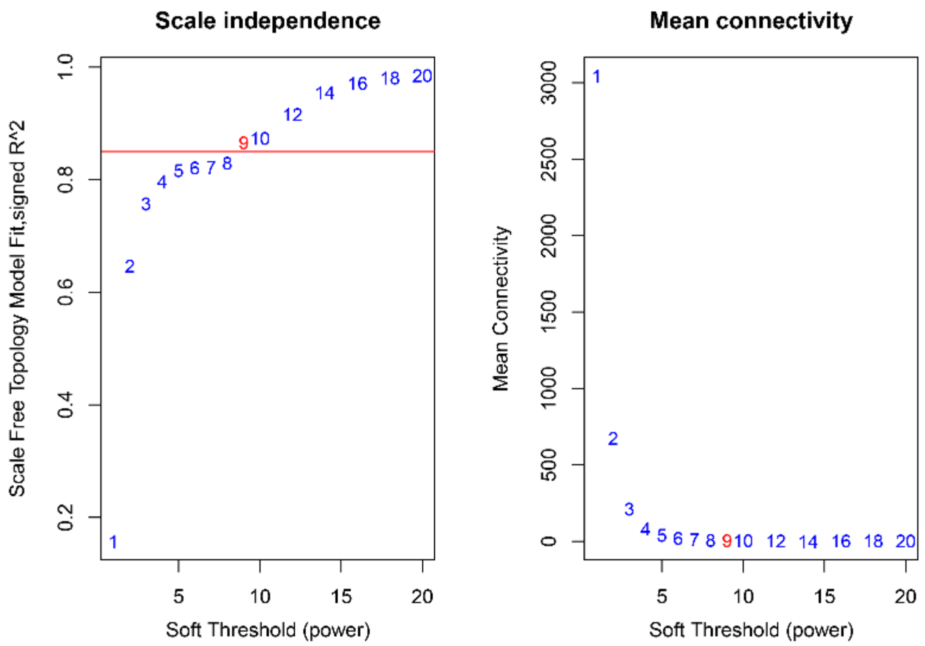

Figure S3. A power of β = 9 was chosen as the optimal soft threshold to ensure a scale-free co-expression network.

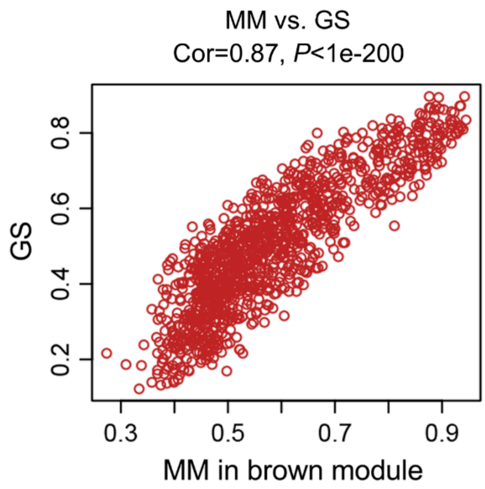

Figure S4. Scatter diagram showed a highly positive correlation between GS and MM in the brown module (r = 0.87, $p < 1 \times 10^{-200}$).

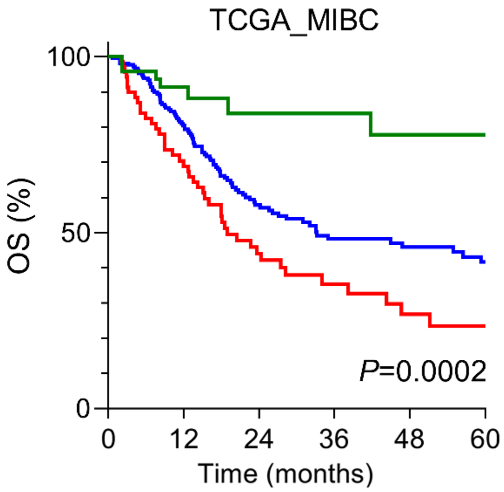

Figure S5. Significant difference of overall survival in TCGA MIBC patients (*P*=0.0002) was observed among different risk subgroups defined by the decision tree.

© 2020 by the authors. Licensee MDPI, Basel, Switzerland. This article is an open access article distributed under the terms and conditions of the Creative Commons Attribution (CC BY) license (http://creativecommons.org/licenses/by/4.0/).
